# *Clostridioides difficile* spores tolerate disinfection with sodium hypochlorite disinfectant and remain viable within surgical scrubs and gown fabrics

**DOI:** 10.1099/mic.0.001418

**Published:** 2023-11-21

**Authors:** Humaira Ahmed, Lovleen Tina Joshi

**Affiliations:** ^1^​ Peninsula Medical School, Faculty of Health, University of Plymouth, Devon, PL4 8AA, UK; ^2^​ Peninsula Dental School, Faculty of Health, University of Plymouth, Devon, PL4 8AA, UK

**Keywords:** biocide, disinfectant, tolerance, spores, *Clostridioides difficile*, transmission

## Abstract

*
Clostridioides difficile* is the most common cause of antibiotic-associated diarrhoea globally. Its spores have been implicated in the prevalence of *C. difficile* infection due to their resistance and transmission ability between surfaces. Currently, disinfectants such as chlorine-releasing agents (CRAs) and hydrogen peroxide are used to decontaminate and reduce the incidence of infections in clinical environments. Our previous research demonstrated the ability of *C. difficile* spores to survive exposure to recommended concentrations of sodium dichloroisocyanurate in liquid form and within personal protective fabrics such as surgical gowns; however, the present study examined the spore response to clinical in-use concentrations of sodium hypochlorite. Spores were exposed to a 10 min contact time of 1000, 5000 and 10 000 p.p.m. sodium hypochlorite, and spore recovery was determined. To understand whether biocide-exposed spores transmitted across clinical surfaces *in vitro*, biocide-exposed spores were spiked onto surgical scrubs and patient gowns and recovery was determined by a plate transfer assay. Scanning electron microscopy was used to establish if there were any morphological changes to the outer spore coat. The results revealed that viable biocide-exposed *C. difficile* spores can be recovered from surgical scrubs and patient gowns, with no observable changes to spore morphology, highlighting the potential of these fabrics as vectors of spore transmission. This study demonstrates that alternative strategies should be urgently sought to disinfect *C. difficile* spores to break the chain of transmission in clinical environments.

## Introduction

*Clostridioides difficile* is a Gram-positive, anaerobic, spore-forming bacterium and the primary aetiological agent of antibiotic-associated diarrhoea globally [1]. Exposure to antibiotics has been shown to disrupt the colonic microbiota of the gut which permits *C. difficile* colonization in 4 % of adults and 15–70 % of infants [2]. Therefore, the spectrum of *C. difficile* infection (CDI) varies from asymptomatic carriage, mild or severe diarrhoea to life-threatening pseudomembraneous colitis, toxic megacolon and death [3, 4]. CDI causes ~29 000 deaths per year in the USA and 8382 deaths per year in Europe, with current data showing that the incidence of CDI was increasing prior to the start of the COVID-19 pandemic in the UK [5, 6]. While the cause of increased CDI during this period is currently a source of debate, these data confirm that *C. difficile* is still very much present within healthcare environments.

Patients with CDI are usually treated with antibiotics such as metronidazole, vancomycin and fidaxomicin [7]. Recent studies, however, have established antibiotic resistance within *C. difficile *; concerningly, plasmid-mediated metronidazole resistance (pMETRO plasmid) has been found and fluoroquinolone-resistant *C. difficile* has been found to be directly associated with metronidazole resistance [8, 9]. Currently research into vancomycin resistance is being explored, and recently *in vivo* resistance to fidaxomicin has been described [10, 11]. The implications of antibiotic resistance in C. *difficile* are significant and include increased infection incidence, mortality and transmission of *C. difficile *. Another factor affecting *C. difficile* spore transmission is the recurrence and reinfection rates in CDI patients [12]. Recurrent CDI (rCDI) occurs after successful antibiotic treatment of CDI where there is either relapse of infection by the same *C. difficile* strain, or reinfection by another strain. The percentage likelihood of rCDI increases significantly after the first episode, with the second recurrence rate at 40 % and subsequent recurrences at 45–65 % [12]. This highlights the urgent need for updated and effective infection control procedures to break the chain of transmission of *C. difficile *.

*C. difficile* produces highly resistant spores with CDI patients able to excrete 10^4^–10^7^ spores per gram of faeces into the environment [13]. Transmission of *C. difficile* occurs primarily in healthcare environments via the faecal–oral route by direct contact with the infected person or by indirect contact with a contaminated source, and once ingested, its spores enter the colon and germinate [1, 4, 14–16]. *C. difficile* spores are highly resistant to biocide disinfectants, desiccation, nutrient depletion, UV light and radiation, meaning they can survive in hostile environments for months [15–17]. Moreover, spores of *C. difficile* spores have been found to be continually transmitted via shoes, surfaces and personal protective equipment (PPE) leading to the continued spread of this infection [18].

Chlorine-releasing agents (CRAs) are used in the disinfection of fluid spills, blood and faeces [19]. Current UK guidelines recommend use of CRAs at 1000 p.p.m. for ~10 min contact time to disinfect surfaces soiled with *C. difficile *; CRAs are a low-cost, efficient disinfection method used in healthcare facilities to disinfect surfaces and include sodium hypochlorite bleach (NaOCl) and sodium dichloroisocyanurate (NaDCC) [19–21]. However, recent studies have shown emerging sporicidal resistance to 1000 p.p.m. NaDCC [21, 22]. The growing evidence of *C. difficile* spore resistance to disinfection with CRAs poses important questions about how to disinfect clinical environments effectively [23]. The mechanisms of CRA action on *C. difficile* spores are poorly understood and current disinfection paradigms are based on studies using *Bacillus* spores [24–28].

In this study, we examined the sporicidal efficacy of sodium hypochlorite disinfectant on *C. difficile* spore survival because we have already conducted similar studies using only NaDCC [21, 22]. We also assessed the transfer ability of *C. difficile* spores from PPE pre- and post-biocide exposure using a plate transfer assay. The PPE tested included surgical scrubs and patient hospital gowns and the presence of spores within fabrics was determined using scanning electron microscopy. This study aimed to increase understanding of the efficacy of NaOCl as a disinfectant and whether direct disinfection of PPE fabrics could potentially limit spore transmission.

## Methods

### Growth conditions, *C. difficile* ribotypes and spore production

*C. difficile* strains from two clinically relevant ribotypes (RTs) were used in this study: the hypervirulent epidemic Stoke-Mandeville R20291 strain (RT 027), clinical isolate DS1813 (RT 027) and the type strain CD630 (RT012). These strains have been widely tested for susceptibility to disinfection [20, 29, 30]. Strains used in this study and their PCR RTs are described in Table 1 and were obtained from the Anaerobic Reference Unit, University Hospital Wales, Cardiff, UK. *C. difficile* cultures from the two RTs were incubated at 37 °C for 48 h in an anaerobic workstation (Don Whitley Scientific) using 85 % nitrogen, 10 % carbon dioxide and 5 % hydrogen gas. Unless otherwise stated, all organisms were stored as spores at 4 °C. All experiments were repeated three times (*n*=3) at room temperature (22 °C).

**Table 1.** *
C. difficile* strains used in the present study [29]

* C. difficile* strain	PCR ribotype	Source
R20291	027	Stoke-Mandeville
DS1813	027	Hinchingbrooke
CD630	012	NCTC

Spores were purified using a previously described method [31, 31]. Briefly, *C. difficile* spores were cultured on reduced Brain Heart Infusion (BHI) agar supplemented with 0.5 % yeast extract, 0.1 % l-cysteine and 0.1 % sodium taurocholate and were examined after 4 days of anaerobic incubation for characteristic colonies. Spores were harvested from the agar plates using Sterile Deionized Water (SDW) and density gradient-washing where spores were centrifugated three times with SDW for 5 min at 10 000 ***g*** and then treated with 50 % HistoDenz (Sigma-Aldrich). Spores were then washed with SDW and stored at 4 °C. Spore purity was confirmed via phase contrast microscopy. Spore concentration was determined via drop count as described by Miles *et al*. [32] and mean colony-forming units (c.f.u.) per ml were calculated [33].

### Exposure of spores to sodium hypochlorite disinfectant

Spores from strains R20291, DS1813 and CD630 (Table 1) at a concentration of 1×10^8^ spores ml^–1^ were independently exposed to 1000, 5000 and 10 000 p.p.m. NaOCl in liquid form for 10 min (recommended contact time) and biocide activity was neutralized with an equal volume of 5 g l^–1^ sodium thiosulphate for 10 min contact time to remove any residual chlorine biocide activity [20, 28, 29]. Spores were enumerated on supplemented BHI agar after anaerobic incubation at 37 °C for 48 h and growth of a single *C. difficile* colony on the agar correlated to evidence of single spore germination [31, 32]. NaOCl (14 % active chlorine; Merck) was diluted to the correct concentration (in p.p.m.) using SDW. For the spore transfer tests to surgical scrubs and patient gowns, the spores were then directly deposited onto each sterile fabric surface and tested via a plate transfer assay (described below). Control experiments where spores were exposed to sodium thiosulphate, sterile deionized water and NaOCl alone were also performed.

### Preparation of PPE fabric surfaces

Multi-use surgical scrubs and patient gowns, worn in UK hospital departments, were ordered from MediSave. Surgical scrubs (order no. VSCRUB) made from 65 % polyester and 35 % cotton, and patient gowns (order no. PG221) made from polycotton were used. To test the transfer of spores to and from the surgical scrubs and patient gowns, the fabrics were aseptically cut into 7×7 cm sections and testing was performed within a drawn circle of 2 cm diameter to conform with the surface area of a 100 g weight which was used to simulate ‘touch’ pressure in this study [22, 33].

### Spore transfer to surgical scrubs and to patient gowns

To determine whether the PPE used in this study was hydrophobic (water repellent) or hydrophilic (attracted to water), and could potentially act as fomites, the surgical scrubs and patient gowns were directly tested with spores in SDW. Spores from strains CD630, DS1813 and R20291 (Table 1) were produced at 1×10^9^ spores ml^–1^ and 100 µl of each was spiked onto each fabric surface and remained in static contact for 10 min before being removed and discarded. If hydrophobic, the liquid would sit on the fabric as a droplet, but if hydrophilic, the time taken for the fabric to absorb all the liquid was recorded. After contact with spores, each section of fabric was aseptically mounted onto a plunger pre-affixed with a 100 g circular weight (to mimic ‘touch pressure’ force) so that the weight was aligned with the test area. An agar plate transfer test was then performed as previously described in Joshi *et al.* [33]. All agar plates were then incubated for 48 h at 37 °C under anaerobic conditions. Following incubation, *C. difficile* colonies were counted and colonies (c.f.u. ml^–1^) were calculated.

### Scanning electron microscopy

Scanning electron microscopy (SEM) was used to determine the presence of characteristic spores before and after treatment with NaOCl. Spores which had not been exposed to NaOCl were used as a comparative control. Samples were fixed with 2.5 % glutaraldehyde and were transferred onto Nucleopore membranes (Sigma-Aldrich) which were sputter coated with gold palladium (60 % Au and 40 % Pd; Testbourne) and argon was used as the sputtering gas. An accelerating voltage of 15 kV was used to view 30 individual spores per sample at ×12 000 magnification (JEOL JSM-6610 Series SEM).

### Statistical analysis

Data are expressed as means±sem. Student t-tests and Kruskal–Wallis tests were performed using Minitab 19 (Minitab), where *P*<0.05 was considered statistically significant.

## Results

### *C. difficile* spores show tolerance to working concentrations of sodium hypochlorite disinfectant

**Fig. 1.**
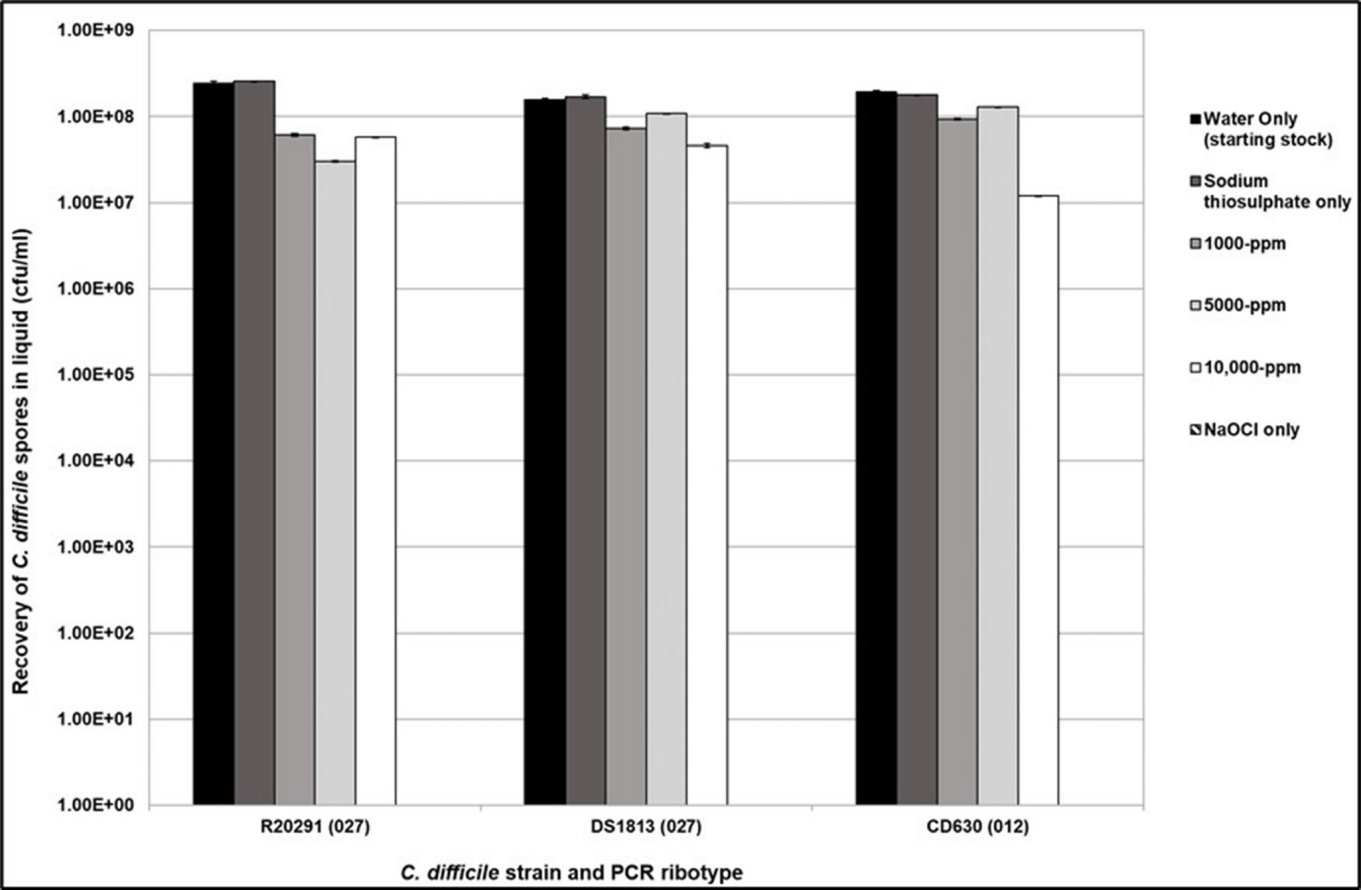
Recovery of purified *C. difficile* spores following exposure to NaOCl at 1000, 5000 and 10 000 p.p.m. in liquid for 10 min. The spore inoculum was at 10^8^ c.f.u. ml^−1^. The inoculum was used as the positive control (water only) and was also suspended in sodium thiosulphate to ensure no cross-reactivity. Plots represent means±sem (*n*=3).

Three clinically relevant strains of *C. difficile* were exposed to in-use working concentrations of sodium hypochlorite (NaOCl) disinfectant at concentrations of 1000, 5000 and 10 000 p.p.m. and biocide activity was neutralized after 10 min contact time. Spores were tested at a concentration of 1×10^8^ c.f.u. ml^−1^. The reduction in spore germination was measured and compared to controls (strains which were not exposed to NaOCl), and spore viability was determined. The results are shown in Fig. 1. Spores from each of the three strains tested showed tolerance to treatment with sodium hypochlorite disinfectant with spore recovery ranging between 1 and 6.5×10^7^ c.f.u. ml^−1^ across all ribotypes. This is a reduction of approximately 1log_10_ (Student’s t-test, *P*<0.05).

Strain R20291 (027) showed an increase in spore recovery following exposure to 10 000 p.p.m. compared to R20291 spores exposed to 1000 and 5000 p.p.m. respectively; however, this was deemed to be non-significant (Kruskal–Wallis, *P*=0.1017). Strain DS1813 (027) showed a 1log_10_ reduction in spores after treatment with NaOCl, with the most effective concentration being 10 000 p.p.m. when compared to the control (Student’s t-test, *P*<0.05). The same is true of CD360 (012) which showed a similar reduction in spore recovery response when exposed to sodium hypochlorite at 10 000 p.p.m. (Student’s t-test, *P*=0.0360). Interestingly, CD630 spore recovery was slightly higher when treated with 5000 p.p.m. NaOCl compared to CD630 spores treated with 1000 p.p.m.; however, this was not significant (Student’s t-test, *P*<0.05).

**Fig. 2.**
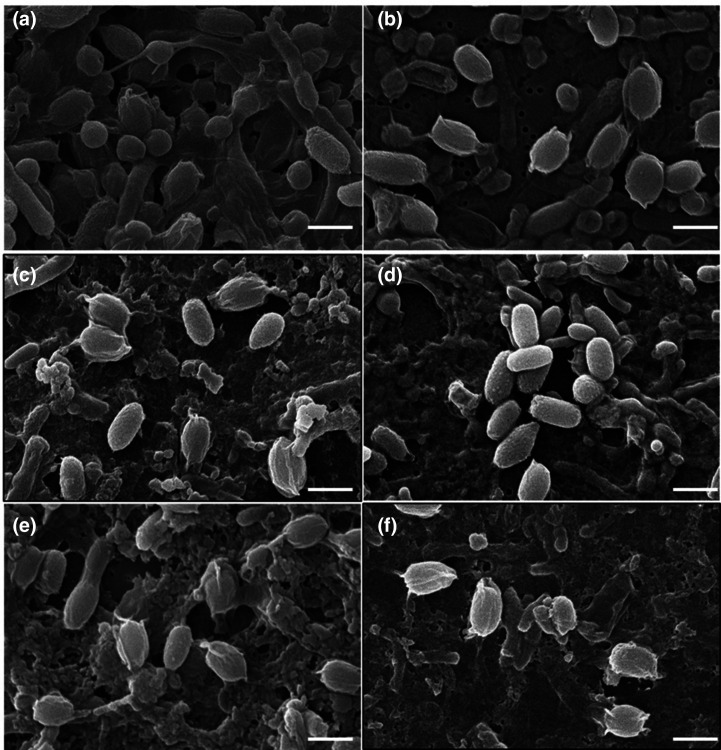
Scanning electron microscopy of *C. difficile* R20291, CD1813 and CD630 spores before and after liquid exposure with 10 000 p.p.m. NaOCl. (**a**) Non-exposed *C. difficile* R20291, (**b**) *C. difficile* R20291 exposed to 10 000 p.p.m. NaOCl, (**c**) non-exposed *C. difficile* DS1813, (**d**) *C. difficile* DS1813 exposed to 10 000 p.p.m. NaOCl, (**e**) non-exposed *C. difficile* CD630 and (**f**) *C. difficile* CD630 exposed to 10 000 p.p.m. NaOCl. Bars, 1 µm in all images. No spore damage is visible following NaOCl exposure. Spores above were imaged at ×12 000 magnification.

SEM was used to visually analyse the surface architecture of *C. difficile* spores before and after 10 000 p.p.m. NaOCl exposure (Fig. 2). SEM studies confirmed the presence of intact spores before treatment; non-treated spores of CD630, DS1813 and R20291 display characteristic oval-shaped spores with a smooth outer surface morphology and evidence of an exosporium (Fig. 2a, c, e) [19, 29]. No visible damage to the spore outer architecture can be seen for any of the strains tested with NaOCl; the post-treatment images show similar morphology to non-treated spores (Fig. 2b, d, f). This supports previous data that spores can tolerate biocide exposure and remain viable.

### Spores adhere to surgical scrubs and patient gowns

**Fig. 3.**
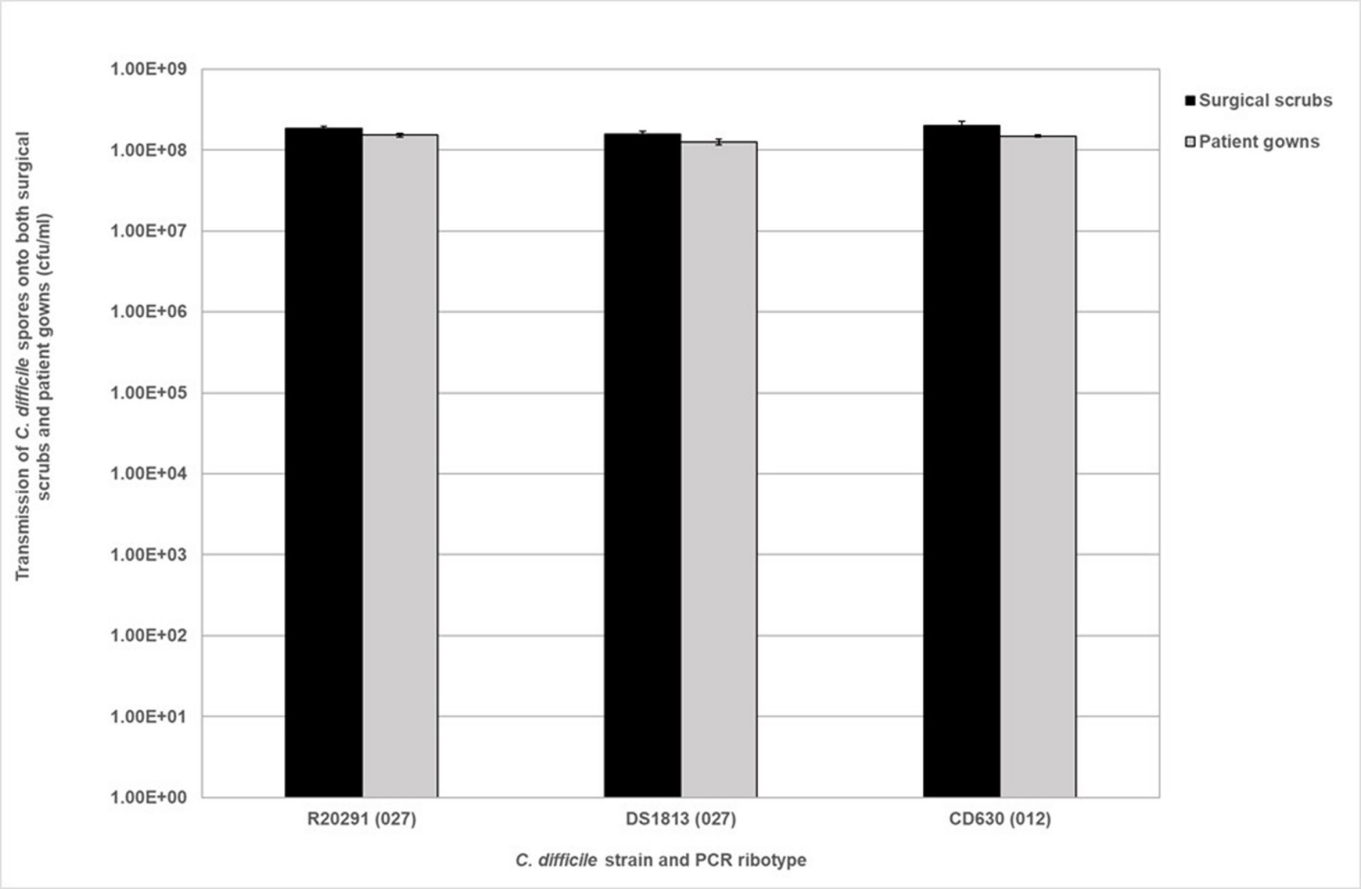
Transmission of purified *C. difficile* spores from spiked surgical scrubs and patient gowns. *C. difficile* spores from strains R20291, DS1813 and CD630 at 1×10^8^ c.f.u. ml^−1^ were inoculated onto the fabrics for a 10 min contact time with each fabric surface. Spores were then recovered via a plate transfer assay. Plots represent mean±sem (*n*=3).

To determine if scrubs and gowns can act as fomites in clinical settings, purified *C. difficile* R20291, DS1813 and CD630 spores were applied at a concentration of 1×10^8^ c.f.u. ml^−1^ to these fabrics for 10 min (Fig. 3). Both fabrics were hydrophilic; surgical scrubs took 30–40 s to absorb the solution while patient gowns took 3 min. In Fig. 3, fewer *C. difficile* spores appear to be recovered from the gowns compared to the scrubs; however, this was not statistically significant, meaning similar amounts of spores adhered and were retained across both fabrics (Kruskal–Wallis, *P*=0.0947). There was also no significant difference in adherence between the strain of *C. difficile* used and the recovery of spores for both scrubs and gowns (Kruskal–Wallis, *P*>0.05).

### Spore transfer to surgical scrubs and patient gowns following exposure to sodium hypochlorite disinfectant

**Fig. 4.**
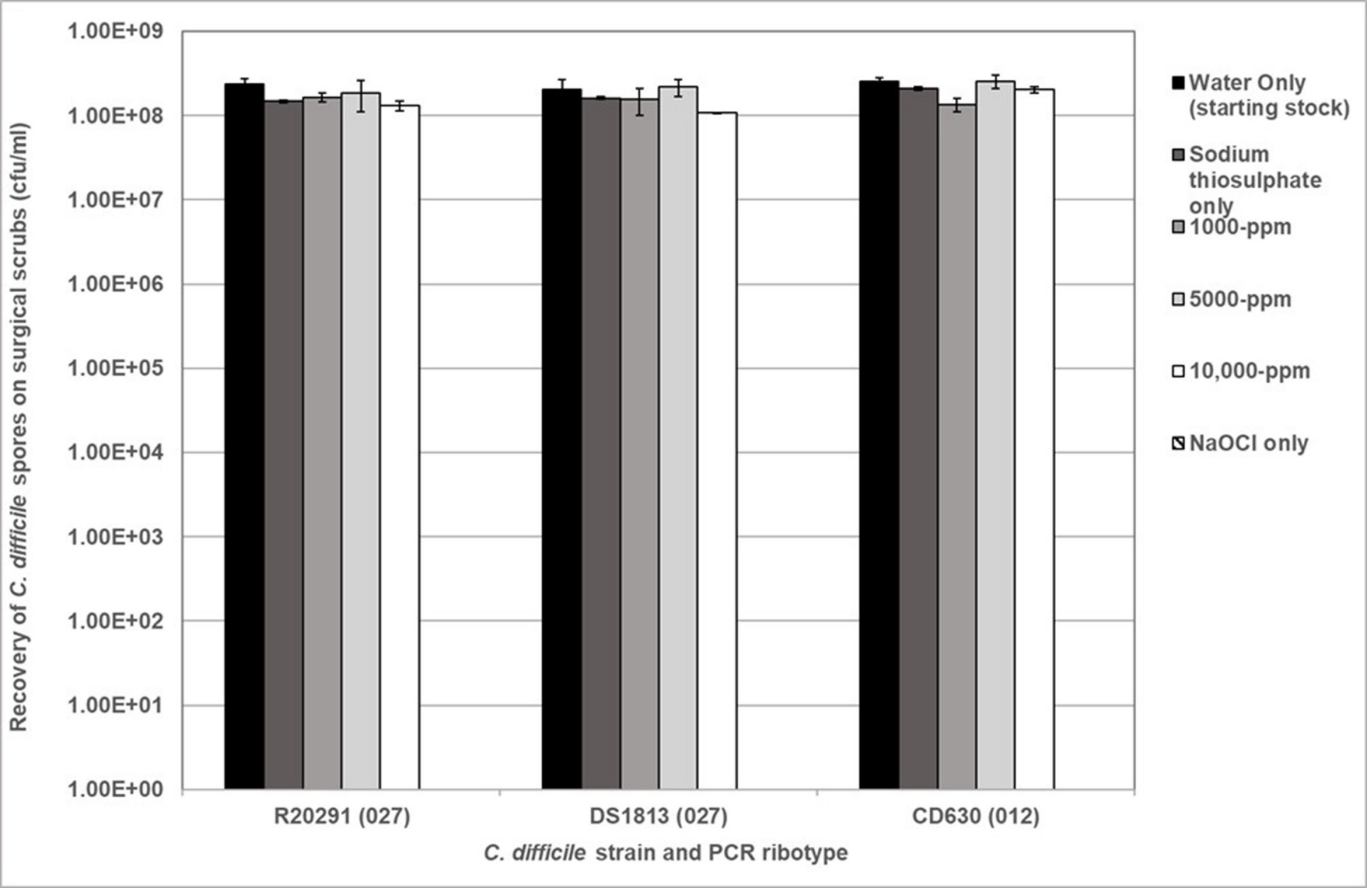
Recovery of purified *C. difficile* R20291, DS1813 and CD630 spores from spiked surgical scrubs following exposure of 10^8^ c.f.u. ml^−1^ to NaOCl at 1000, 5000 and 10 000 p.p.m. for 10 min. Spores not exposed to NaOCl (water only) were used as a positive control and the use of NaOCl only was used as a negative control, where no spores were recovered. Plots represent mean±sem (*n*=3).

Previously, spore survival was determined in liquid (Fig. 1) following treatment with NaOCl. To determine whether spores transferred after exposure to biocide, *C. difficile* spores were inoculated onto surgical scrubs and treated with NaOCl (Fig. 4). *C. difficile* R20291 spore recovery from scrubs decreases after exposure to 1000, 5000 and 10 000 p.p.m. NaOCl compared to non-exposed R20291 spores, indicating they are retained on the fabric with no significant difference between strains and biocide concentration (Student’s t-test, *P*>0.05). *C. difficile* DS1813 spores demonstrate no significant reduction in spore recovery following treatment with 1000, 5000 and 10 000 p.p.m. NaOCl (Kruskal–Wallis, *P*>0.05). In Fig. 4, a clear reduction in *C. difficile* CD630 spore recovery is displayed after 1000 and 5000 p.p.m. NaOCl treatment compared to non-exposed CD630 spores, but this was not significant (Kruskal–Wallis, *P*>0.05). A 1log_10_ reduction in CD630 spore recovery is seen between non-exposed and 10 000 p.p.m. NaOCl-treated but no statistical significance was determined (Student’s t-test, *P*=0.0777).

**Fig. 5.**
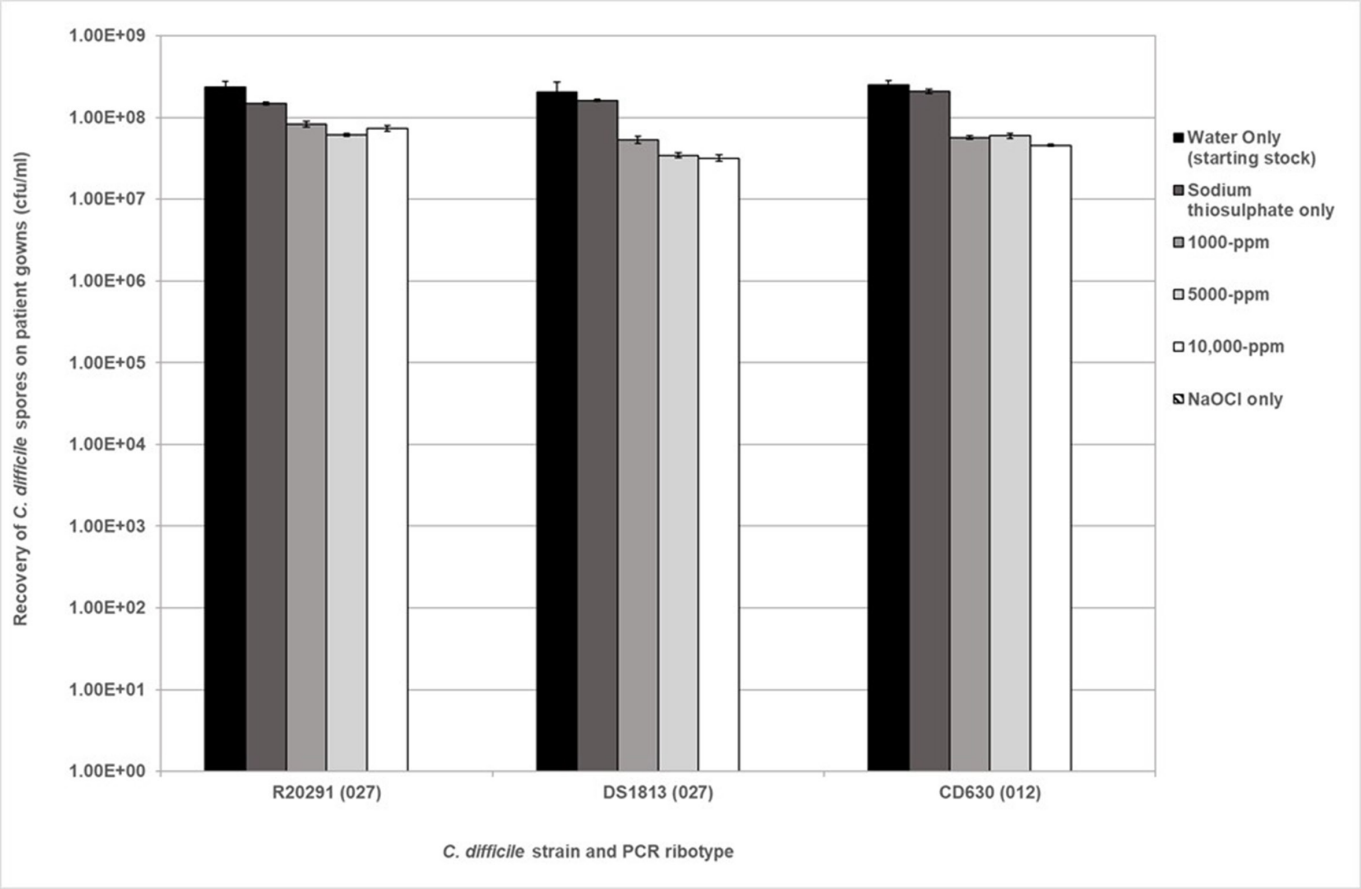
Recovery of purified *C. difficile* R20291, DS1813 and CD630 spores from spiked patient gowns following exposure of 10^8^ c.f.u. ml^−1^ to NaOCl at 1000, 5000 and 10 000 p.p.m. for 10 min. Spores not exposed to NaOCl (water only) were used as a positive control and the use of NaOCl only was used as a negative control, where no spores were recovered. Plots represent mean±sem (*n*=3).

*C. difficile* spore recovery was then determined from patient gowns (Fig. 5). An approximate 1log_10_ reduction in R20291 spore recovery was observed between non-exposed and NaOCl-exposed R20291 spores, although no significant difference in spore recovery was identified, indicating spores remained adhered to the gowns (Kruskal–Wallis, *P*>0.05). In Fig. 5, it appears there is an increase in R20291 spore recovery following 10 000 p.p.m. NaOCl exposure compared to R20291 spores exposed to 5000 p.p.m. NaOCl but no significant difference was found (Student’s t-test, *P*=0.3168). DS1813 spore survival seems to decrease as NaOCl concentration increases, but no significance was detected (Kruskal–Wallis, *P*>0.05). A 1log_10_ reduction was detected between non-exposed CD630 spores and CD630 spores exposed to all NaOCl concentrations (Student’s t-test, *P*<0.05).

## Discussion

Management of CDI in healthcare settings relies on effective infection control measures to reduce the spread of *C. difficile* spores [34]. This study explored the efficacy of NaOCl for disinfection of *C. difficile* spores in liquid, and on clinically relevant fabrics including surgical scrubs and patient gowns. Currently, the National Health Services use a working concentration of 1000–5000 p.p.m. of chlorine for 10 min for spore decontamination on surfaces [19]. However, in this study 1000 p.p.m. NaOCl only reduced *C. difficile* R20291 spore recovery in liquid compared to the other strains tested (Figs 1, 3 and 4). Interestingly, the hypervirulent R20291 and DS1813 (RT027) strains (Table 1) have been suggested to be more resistant to CRAs compared to other ribotypes [20, 22, 29, 30] supporting our data. When spores were treated with 10 000 p.p.m. NaOCl in liquid there was low log reduction, but the literature indicates that the most effective disinfectants yield a log reduction of between 3 and >5 log units generally [30, 35, 36]. Previous biocide contact studies have found similar results when exploring *C. difficile* spore killing with CRAs [29, 30, 37].

Most hospital-acquired infections are transmitted through the hands of healthcare workers and by direct contact with fomites [18]. Consequently, scrubs and gowns are worn in hospitals as PPE to reduce the transmission of infectious material and break the chain of transmission. Previously, we showed that surgical single-use gown ties, when worn inappropriately, can act as fomites and contribute to transmission of *C. difficile* in clinical environments [18]. This present study highlights that both surgical scrubs and patient gowns can act as fomites as they attracted and retained *C. difficile* spores which could then be transferred to a hydrophilic surface (Figs 3–5). This supports the results from similar studies, where fabrics and surgical scrubs retained *Pseudomonas aeruginosa* and methicillin-resistant *Staphylococcus aureus* bacteria [38, 39].

Differences in the physiochemical properties of scrubs and gowns have been shown to play a critical role in the dissemination of microorganisms through hospitals [37, 40]. The limited variation in R20291, DS1813 and CD630 spore recovery from these fabrics may be due to spore hydrophobicity; these strains have hydrophobicity values of 62, 77 and 63 %, respectively [33]. Fewer *C. difficile* spores were recovered from the fabrics (Fig. 3) than liquid (Fig. 1), suggesting spores may survive better within the fabrics by adhering to specific fibres. This adherence result correlates with our previous studies that tested NaDCC on clinically relevant fabrics, showing more spores remained on the fabric fibres, and hence the ability of the spores to transfer over to the hydrophilic agar plate was reduced [21, 22]. The strains of spores tested in this study were also resistant to NaOCl decontamination directly on the surgical scrubs, indicating these spores can remain viable, adhere to and subsequently transfer onto different clinical surfaces. Similarly, the survival of spores on these fabrics after exposure to NaOCl suggests that the biocide may not have penetrated the PPE effectively to interact directly with the spores. This is useful in some respect given that PPE is designed to be hydrophobic; however, in the case of our study, we have already shown that these fabrics attract spores within liquid [22]. This emphasizes the need to use appropriate disinfection measures when disinfecting PPE such as surgical scrubs and patient gowns [18].

In this study, SEM was used to determine whether CRA use physically damaged the outer spore layers. The results clearly demonstrated no visible effect on spore morphology after biocide exposure, which correlates with results from our previous study [20]. *C. difficile* spores characteristically possess an exosporium layer that is closely associated with spore adherence to organic and inorganic surfaces. Damage to the outer exosporium layer and spore coat has been hypothesized to change the adherence and transmissibility of spores [33]. In this study, the lack of damage to the *C. difficile* spores highlights increased tolerance of clinically relevant spores to disinfection with CRAs, and their increased adherence ability to clinical fabrics after biocide exposure (Figs 3–5). These results suggest that CRA use within clinical settings should be evaluated to ensure that efficaeous disinfection methods are employed to reduce the potential for spore transmission and CDI. Indeed, more NHS trusts are using alternative means of disinfection such as hydrogen peroxide fogging and UV-C treatment to effectively manage CDI [41–43]. It is noted, however, that in low- to middle-income countries (LMICs) the use of cheaper and accessible biocides such as CRA are essential in infection control [44]. The impact of CRA tolerance in microorganisms within LMIC healthcare settings has yet to be elucidated but understanding biocide tolerance within these settings could be an important factor in manging the emergence of antimicrobial-resistant bacteria.

Effective management of CDI focuses on ensuring hospital infection and control practices are being conducted appropriately [6]. This includes preparing CRAs fresh on the day to ensure optimal activity and to disinfect surfaces that a soiled scrub or gown may come into contact with twice daily [45, 46]. Antimicrobial surface wiping techniques also need to be explored as mechanically removing spores by wiping surfaces has been found to be effective in reducing spore transmission [46]. Spore inactivation at varying temperatures also needs to be explored to determine optimal laundering conditions for these fabrics, as *C. difficile* spores have been found to survive the NHS laundering process [47]. *Bacillus subtilis* spores have been effectively inactivated with moist heat at >100 °C, which may be useful for *C. difficile* spore inactivation [44]; however, the effects of laundering on multi-use protective clothing has sparked controversy as high laundering temperatures could destroy hydrophobic PPE fabrics, reducing their ability to prevent *C. difficile* transmission [48].

In this study we have demonstrated that spores of different clinical *C. difficile* strains can survive exposure to concentrations of sodium hypochlorite. There is no significant reduction in spore viability which demonstrates that disinfection with CRAs is not effective. Previous studies have only rigorously tested *Bacillus* species against CRAs *in vitro* using bacterial kinetics; however, limited studies have sought to examine the effectiveness of CRAs against clinically relevant *C. difficile* spores [24–28]. This study seeks to address the gaps in the literature surrounding *C. difficile *-specific spore responses to CRA biocides. Therefore, it is currently unclear whether the tolerance of *C. difficile* spores is an observational phenomenon, or whether evolution is driving CRA tolerance at a genomic level. It is possible that factors such as temperature, exposure time and pH may affect the efficacy of CRA action, and this should be further explored to fully understand *C. difficile* resistance to CRAs.

## Conclusion

This study highlights the ability of *C. difficile* spores to tolerate NaOCl disinfection at in-use recommended active chlorine concentrations. Understanding the molecular basis of these interactions is integral to practical management of CDI and reducing the burden of infection in healthcare settings. Unanswered questions remain regarding the extent of biocide tolerance within *C. difficile* and whether biocide tolerance is affected by antibiotic co-tolerance. Currently, this is important given that antimicrobial resistance is increasing globally. There is an urgent need to review current disinfection and infection prevention guidelines to optimize *C. difficile* spore disinfection practices and reduce the incidence of CDI globally.
